# Trends, influencing factors and prediction analysis of under-five and maternal mortality rates in China from 1991 to 2020

**DOI:** 10.3389/fpubh.2023.1198356

**Published:** 2023-10-19

**Authors:** Meng Zhang, Huimin Qu, Junfen Xia, Xiaoqing Hui, Cannan Shi, Feng Xu, Junjian He, Yuan Cao, Mengcai Hu

**Affiliations:** Department of Health Care, The Third Affiliated Hospital of Zhengzhou University, Zhengzhou, Henan, China

**Keywords:** ARIMA, linear mixed effect model, maternal mortality rate, trends, under-five mortality rate

## Abstract

**Introduction:**

Under-five mortality rate (U5MR) and maternal mortality rate (MMR) are important indicators for evaluating the quality of perinatal health and child health services in a country or region, and are research priorities for promoting maternal and infant safety and maternal and child health. This paper aimed to analysis and predict the trends of U5MR and MMR in China, to explore the impact of social health services and economic factors on U5MR and MMR, and to provide a basis for relevant departments to formulate relevant policies and measures.

**Methods:**

The JoinPoint regression model was established to conduct time trend analysis and describe the trend of neonatal mortality rate (NMR), infant mortality rate (IMR), U5MR and MMR in China from 1991 to 2020. The linear mixed effect model was used to assess the fixed effects of maternal health care services and socioeconomic factors on U5MR and MMR were explored, with year as a random effect to minimize the effect of collinearity. Auto regressive integrated moving average models (ARIMA) were built to predict U5MR and MMR from 2021 to 2025.

**Results:**

The NMR, IMR, U5MR and MMR from 1991 to 2020 in China among national, urban and rural areas showed continuous downward trends. The NMR, IMR, U5MR and MMR were significantly negatively correlated with gross domestic product (GDP), the proportion of the total health expenditure (THE) to GDP, system management rate, prenatal care rate, post-natal visit rate and hospital delivery rate. The predicted values of national U5MR from 2021 to 2025 were 7.3 ‰, 7.2 ‰, 7.1 ‰, 7.1 ‰ and 7.2 ‰ and the predicted values of national MMR were 13.8/100000, 12.1/100000, 10.6/100000, 9.6/100000 and 8.3/100000.

**Conclusion:**

China has made great achievements in reducing the U5MR and MMR. It is necessary for achieving the goals of Healthy China 2030 by promoting the equalization of basic public health services and further optimizing the allocation of government health resources. China’s experience in reducing U5MR and MMR can be used as a reference for developing countries to realize the SDGs.

## Introduction

Women and children make up the two-thirds of the world’s population. Thus, the health of them not only exerts a strong influence on their personal and family happiness but also provides a basic premise for the sustainable development of mankind and, even more, a comprehensive index for the development of social economy and human progress (1, 2). As the important indicators to assess the service quality of national and regional perinatal care and child-care, the under-five mortality rate (U5MR) and maternal mortality rate (MMR) were the research focus for promoting the safety and health of children and women (2, 3).

According to the Millennium Development Goals (MDGs), which was formulated at the United Nations Summit in September 2000, the goals to reduce child mortality rate by two-thirds and maternal by three quarters were scheduled to be fulfilled in 2015 (4–6). The United Nations Sustainable Development Goals (SDGs) are 17 global development goals set by the United Nations to guide global development efforts from 2015 to 2030 beyond the expiration of MDGs from 2000 to 2015. Target 3 (good health and well-being) aims to reduce the global MMR to 70/100,000 and U5MR to 25‰ by 2030. Put forward by the Chinese government, outline of the Healthy China 2030 plan points out that, in the year of 2030, the infant mortality rates (IMR), U5MR and MMR will reduce to 5.0 ‰, 6.0 ‰ and 12.0/100000, respectively, (7). Over the past decades, China has made intense efforts in various ways to enhance the life quality of women and children. Besides, it is the U5MR and MMR that shows a downward trend with each passing year (8, 9). However, there are still plenty of challenges to face for the maternal and child health services. First of all, with the implementation of “Universal Two-child Policy” and the increase of high-risk pregnant women, the absolute number of maternal and child deaths is large, and which is difficult to present a continuous momentum of decline (10, 11). Apart from that, the obvious gap between urban and rural areas also poses a formidable barrier to further reduce the mortality. Additionally, the health-care level of maternal and child health care in China, especially in rural areas, still have gap with the developed countries (12, 13).

Simple as the maternal and child health indicators seem to be, they are the typical reflection of the economy, culture and policy in a country or region (14, 15). Currently, what is still unclear is to what degree these contributing factors, such as social health care services, the social economic, the total health expenditure (THE) and so on, can affect the U5MR and MMR. This paper aimed to present the U5MR and MMR, demonstrate the trends to change with time and explore the influence caused by social health-care and economic on the U5MR and MMR, about which this research also aimed to make a prediction to provide the scientific foundation for improving the quality of maternal and child health services and to supply reference for relevant policies and measures of maternal care formulated by certain departments to further reduce the mortality rates.

## Methods

### Data sources

The data used in this study including the data of neonatal mortality rate (NMR), IMR, U5MR, MMR, maternal system management rate, prenatal care rate, postnatal visit rate and hospital delivery rate from 1991 to 2020 were collected from the annual China Health Statistics Yearbooks (1991–2021). The data of GDP, and total health expenditure (the ratio of total health expenditure (THE) to GDP) from 1991 to 2020 were collected from official data released by the National Bureau of Statistics of China (16). The data above were shown in Additional Files 1, 2.

### Definitions

NMR refers to the number of neonatal deaths that from birth to 28 days per 1,000 live births. IMR is defined as the number of infants deaths that from birth to the 1 year old per 1,000 live births. U5MR refers to the number of deaths of children under 5 years of age per 1,000 live births. MMR is defined as the number of the death caused by any pregnancy or pregnancy treatment within 42 days from pregnancy to postpartum per 100,000 live births. The number of live births are defined as the number of newborns with one of the four vital signs of heartbeat, respiration, umbilical cord fluctuation and voluntary muscle contraction after delivery after 28 weeks of pregnancy and above.

### Statistical analysis

The JoinPoint regression model was established by JionPoint 4.9.1.0 software to analyze the change trend of IMR, U5MR and MMR in China from 1991 to 2020. The data of annual percent change (APC) and average annual percent change (AAPC) were the main outcome indicators of the JoinPoint model, both of which represent the percentage change of variables with the year. The former was used to evaluate the internal trend of each segment interval, and the latter was used to evaluate the overall change trend. Joinpoint pairwise comparison test was applied to compare the trend differences between urban and rural areas. The linear mixed effect model was used to explore the fixed effect of socioeconomic factors and maternal health care level on IMR, U5MR and MMR, and the year was used as a random effect to reduce the effect of col-linearity, and the socioeconomic indicators were logarithmically converted. The auto regressive integrated moving average (ARIMA) model was built using SPSS 21.0 for time series prediction. ARIMA (p, d, q) combines autoregressive analysis (AR) and moving average (MA) including three parameters: the order of AR (p), the degree of difference (d), the order of MA (q). The national data from 1991 to 2015 was used for modeling, and the data from 2016 to 2020 for evaluating the mean absolute percentage error (MAPE) of models, and the optimal model was applied to predict the rates from 2021 to 2025. A two-side *p* of <0.05 was considered significant in this study.

## Results

### Trends analysis results

**Figure 1:**
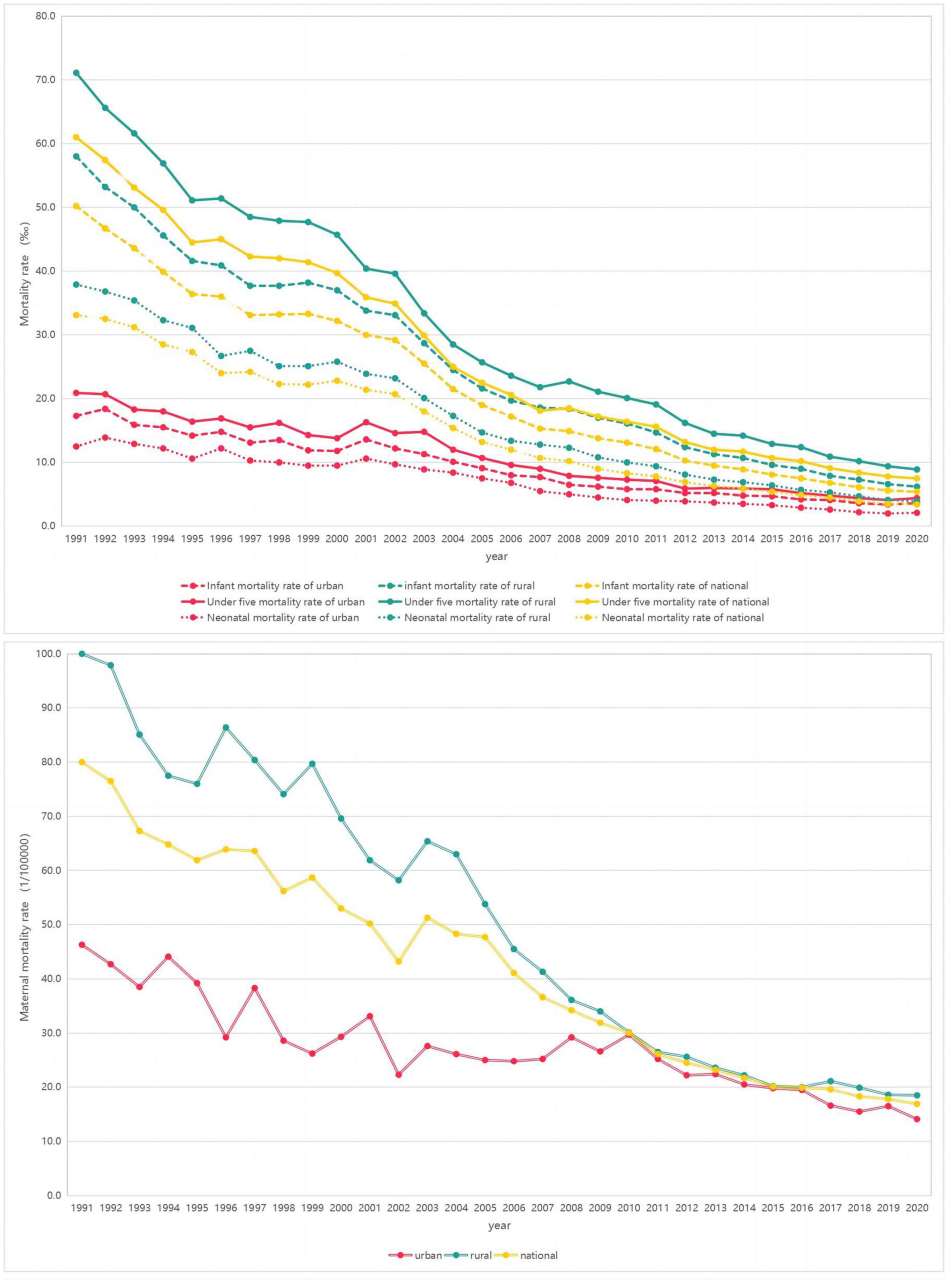
Trends of the NMR, IMR, U5MR and MMR from 1991 to 2020 of China in national, urban and rural areas.

The NMR, IMR, U5MR and MMR from 1991 to 2020 in China among national, urban and rural areas showed continuous downward trends (Figure 1). Table 1 shows the results of APC and AAPC reflecting the change trend of NMR, IMR, U5MR and MMR in the past 30 years. The results of JoinPoint pairwise comparison test showed that the AAPC differences of NMR, IMR, U5MR and MMR between urban and rural areas from 1991 to 2020 were 1.4% (95%CI = -0.7, 3.6%, *p* = 0.193), 1.8% (95%CI = 0.8, 2.8%, *p* < 0.001), 1.6% (95%CI = 0.5, 2.7%, *p* = 0.003) and 2.1% (95%CI = 0.1, 3.1%, *p* = 0.005), respectively. The data above were shown in Additional File 3.

**Table 1:** JoinPoint analysis of infant mortality rate, under five mortality rate and maternal rate in national, urban and rural areas.

	Trend 1		Trend 2		Trend 3		AAPC (%)	95% CI for AAPC (%)
	Years	APC (%)	Years	APC (%)	Years	APC (%)		
National	1991–2002	−4.3	2002–2005	−12.9	2005–2020	−8.8	−7.6	(−8.5, −6.6)
Urban	1991–2004	−3.2	2004–2007	−13.8	2007–2020	−7.4	−6.2	(−8.1, −4.3)
Rural	1991–2002	−4.5	2002–2005	−11.7	2005–2020	−8.7	−7.4	(−8.5, −6.3)
National	1991–2002	−4.5	2002–2005	−12.3	2005–2020	−8.2	−7.3	(−8.3, −6.2)
Urban	1991–2003	−3.5	2003–2008	−10.0	2008–2020	−5.4	−5.5	(−6.3, −4.6)
Rural	1991–1997	−6.7	1997–2000	−0.7	2000–2020	−8.6	−7.4	(−8.6, −6.2)
National	1991–2002	−4.7	2002–2005	−13.3	2005–2020	−7.1	−6.9	(−8.0, −5.8)
Urban	1991–2003	−3.1	2003–2008	−10.2	2008–2020	−5.3	−5.3	(−6.2, −4.3)
Rural	1991–2002	−4.9	2002–2005	−11.8	2005–2020	−7.0	−6.7	(−8.0, −5.4)
National	1991–2005	−3.7	2005–2013	−8.2	2013–2020	−4.0	−5.0	(−5.7, −4.4)
Urban	1991–2002	−5.2	2002–2010	0.9	2010–2020	−6.2	−3.9	(−5.2, −2.7)
Rural	1991–2004	−3.6	2004–2012	−10.8	2012–2020	−3.3	−5.6	(−6.4, −4.8)

### Linear mixed model results

The results of linear mixed model (Table 2) showed that NMR, IMR, U5MR and MMR were significantly negatively correlated with GDP, the proportion of THE to GDP, system management rate, prenatal care rate, post-natal visit rate and hospital delivery rate (*p* < 0.05).

**Table 2:** The results of linear mixed model.

	Neonatal mortality rate		Infant mortality rate		Under five mortality rate		Maternal mortality rate	
	*β*	95%CI	*β*	95%CI	*β*	95%CI	*β*	95%CI
GDP^#^	−19.392	(−20.422, −18.362)	−27.386	(−28.808, −25.964)	−33.045	(−35.176, −30.914)	−38.599	(−40.607, −36.590)
Proportion of THE in GDP	−8.625	(−10.836, −6.414)	−12.057	(−15.246, −8.869)	−14.499	(−18.432, −10.567)	−17.729	(−21.776, −13.682)
System management rate	−0.854	(−1.038, −0.671)	−1.206	(−1.464, 0.949)	−1.450	(−1.773, −1.128)	−1.932	(−2.189, −1.674)
Prenatal care rate	−1.157	(−1.358, −0.957)	−1.598	(−1.883, −1.313)	−1.938	(−2.287, −1.588)	−2.244	(−2.671, −1.817)
Postpartum visit rate	−1.242	(−1.437, −1.048)	−1.720	(−1.994, −1.446)	−2.081	(−2.422, −1.740)	−2.456	(−2.832, −2.079)
Hospital delivery rate	−0.530	(−0.566, −0.494)	−0.746	(−0.801, −0.691)	−0.907	(−0.968, −0.847)	−1.039	(−1.139, −0.939)

GDP, Gross Domestic Product; THE, total health expenditure.

### ARIMA models

This SPSS21.0 software was used to fit ARIMA prediction models with NMR, IMR, U5MR and MMR as dependent variables. Because most of the above time series were non-stationary, the series were stabilized after one difference transformation. The results of the selected optimal model, R^2^, BIC and MAPE were shown in Table 3. All of the residual sequences were white noise sequence with the *p* values of Ljung-BoxQ tests greater than 0.05.

**Table 3:** The results of ARIMA models.

	ARIMA	R^2^	BIC	MAPE
Neonatal mortality rate				
National	(1,1,2)	0.994	0.038	3.635%
Urban	(3,1,3)	0.966	0.480	6.346%
Rural	(3,1,1)	0.992	0.714	3.849%
Infant mortality rate				
National	(0,1,0)	0.993	0.398	3.387%
Urban	(2,1,2)	0.973	0.306	4.533%
Rural	(1,1,0)	0.993	0.862	3.677%
Under five mortality rate				
National	(0,1,2)	0.993	1.131	3.963%
Urban	(3,1,4)	0.973	1.071	4.752%
Rural	(2,1,0)	0.991	1.611	3.902%
Maternal mortality rate				
National	(1,1,3)	0.978	2.883	4.696%
Urban	(3,0,1)	0.850	3.264	8.624%
Rural	(3,1,4)	0.973	4.281	5.704%

### Prediction results of ARIMA models

**Figure 2:**
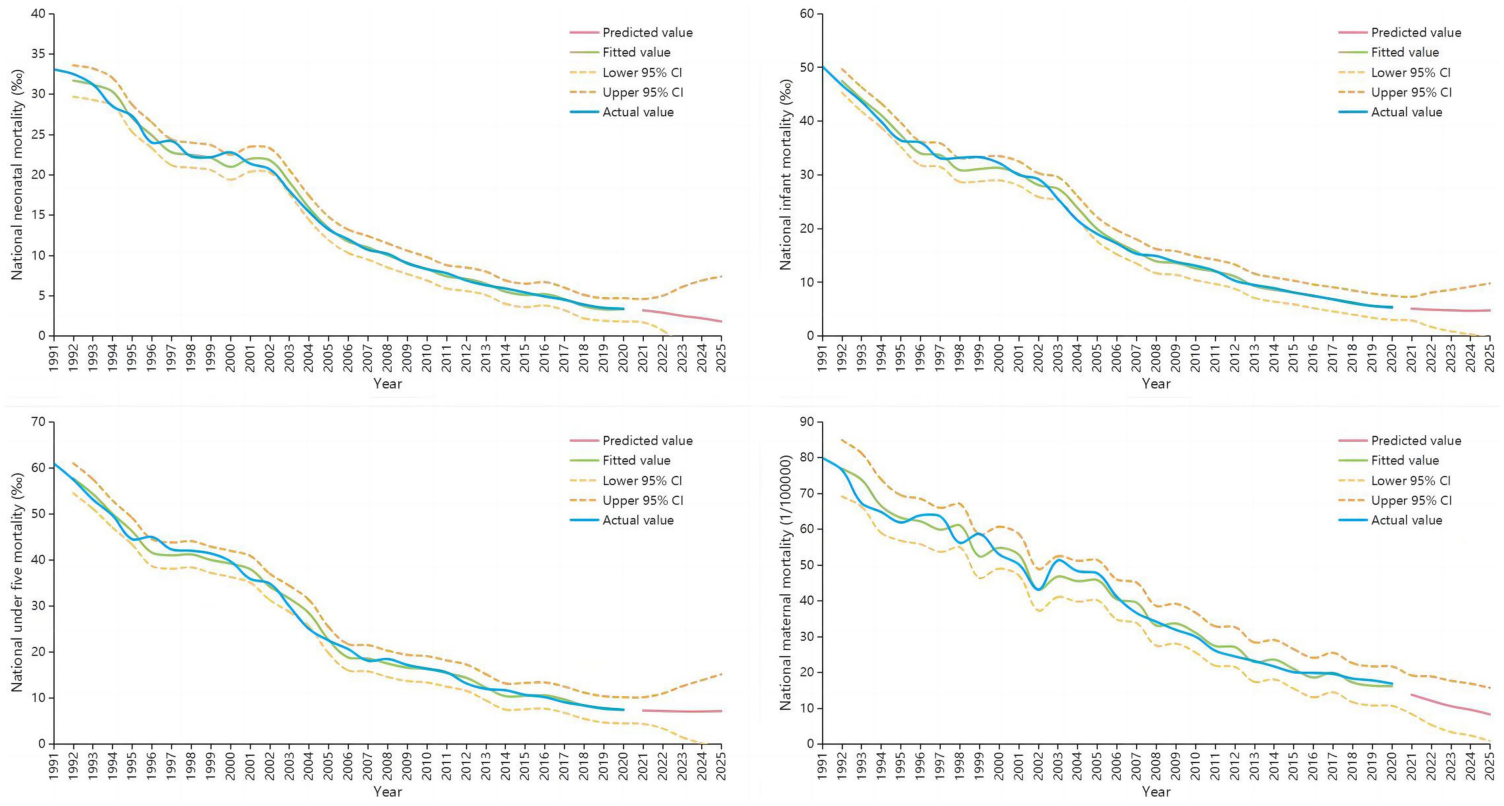
The predicted results of ARIMA model.

The predicted results of ARIMA model were shown in Figure 2 and Additional File 3. The predicted values of national NMR in from 2021 to 2025 were 3.2 ‰, 2.9 ‰, 2.5 ‰, 2.2 ‰ and 1.8 ‰ respectively. The predicted values of the national IMR were 5.1 ‰, 4.9 ‰, 4.8 ‰, 4.7 ‰ and 4.8 ‰ respectively. The predicted values of the national U5MR are 7.3 ‰, 7.2 ‰, 7.1 ‰, 7.1 ‰ and 7.2 ‰. The predicted values of national MMR are 13.8/100000, 12.1/100000, 10.6/100000, 9.6/100000 and 8.3/100000. The predicted results of urban and rural areas were shown in the Additional File 4.

## Discussion

The NMR, IMR, U5MR and MMR of China were on the decline from 1991 to 2020, and the rates in rural areas has been higher than that in urban areas, which might be caused by the imbalance of economic development and health services (17, 18). The annual average decline rates of NMR, IMR, U5MR and MMR in rural areas were greater than that in urban areas, and the differences were reduced between rural and urban, which showed that the maternal health care strategy of China, especially in rural areas, has made remarkable progress, and the quality of maternal medical services has improved (19–21), which may be related to the development of public health services projects and the launch of basic public health services equalization projects (22, 23). In addition to increased accessibility to healthcare services, this might be related to rapid economic growth and improved transportation (24).

Since 1990, the health level of Chinese children has improved significantly, and the MDGs were achieved 8 years ahead of schedule in 2007. China’s global ranking of U5MR dropped from 90th in 1990 to 133rd in 2019 (25). Currently, some progress has been made in reducing child mortality in China, but concerted efforts are still needed to avoid preventable under-five deaths in the future. Studies have shown that the distribution of causes of death varies between age groups, thus requiring preventive interventions targeting specific age groups (26, 27). Congenital diseases and accidental death account for a large proportion of under five children deaths (28–30), and it is recommended to conceive at the appropriate age, make prenatal diagnosis, improve the safety awareness and safety protection skills of the whole society (31, 32).

At present, obstetric hemorrhage is still one of the leading causes of maternal death in China (33). Prenatal care, skilled delivery, obstetric emergency care and postnatal care can effectively reduce the risk of obstetric hemorrhage. Therefore, improving the hospital delivery rate can also be explained by an effective reduction in MMR, which was also consistent with the mixed linear model results of this study. With the adjustment of China’s birth policy, the proportion of older adult and high-risk pregnant women may continue to rise, and the control of maternal deaths in the whole country and all regions is facing great challenges (34). In addition, the capacity building of medical institutions and the pregnancy and perinatal health care management of the floating population should be strengthened to ensure the safety of mothers and infants and further reduce MMR.

The results of linear mixed model suggest that NMR, IMR, U5MR and MMR were negatively correlated with GDP, the proportion of THE in GDP, system management rate, prenatal care rate, postpartum visit rate and hospital delivery rate, suggesting that the development of economic level, the investment of THE and the improvement of maternal and child health care management level would help to further reduce NMR, IMR, U5MR and MMR. Therefore, the health of children and pregnant women in poor areas should get more attention, and the relevant government departments should formulate corresponding policies and measures to implement the medical assistance system for children and pregnant women in poor areas, and effectively reduce the U5MR and MMR.

The ARIMA prediction results were that the national NMR, IMR, U5MR and MMR would be 1.8 ‰, 4.8 ‰, 7.2 ‰ and 8.3/100000 in 2025, which can meet the goals set out in the Health China 2030 planning outline (9). However, the results also showed that the rate of decline has decreased, and even in 2025, IMR and U5MR have increased from the previous year, which may be due to the following three reasons. Firstly, after IMR and U5MR have fallen to a certain level, the rate of decline may decrease. A study of IMR in Scotland showed that it had fallen from 5.75 ‰ in 2000 to 3.25 ‰ in 2018, a much slower rate of decline than the average rate of decline in China over the past 30 years (35). Secondly, the mortality rates predicted in this study are getting closer to those of developed countries. Studies have shown that IMR in Canada and the United States are 4.7 and 4.2, respectively (36, 37). However, the level of maternal and child health in rural areas of China still lags behind developed countries. Therefore, focusing on improving maternal and child health care level in rural areas remains a key factor in improving the health and quality of life of Chinese women and children. Third, few studies have analyzed the main causes and changing trends of IMR and U5MR in China. One study showed that the increase in IMR in the England since 2014 was mainly due to an increase in early NMR among preterm babies born at less than 24 weeks gestation (38). This also suggests that the causes of death in U5MR in China need to be further assessed. The predicted results in rural areas of this study were that the IMR, U5MR, and MMR would be 6.0 ‰, 8.6 ‰ and 14.0/100000, respectively, in 2025, which is higher than that in urban areas. Although the gap between urban and rural areas has narrowed, the rate of decline in rural areas is slower than in urban areas, which is also suggested that relevant departments should improve policies, strengthen the training of professional skills of grass-roots medical staff, and strive to improve the quality of rural medical and health services.

Our study has some strength. We analyzed the changing trends of long-term U5MR and MMR in China and made predictions, providing the basis for relevant departments to develop relevant child and maternal health care policies and measures. This study assessed the impact of social health services and economic factors on U5MR and MMR, which have been seldom evaluated in previous studies. This study also has some limitations, the data used in this study were from the surveillance systems and the data quality might be inconsistent in different areas such as urban and rural areas. The ARIMA model was suitable for the short-term prediction model and could not achieve the long-term trend prediction. Therefore, it is necessary to continuously collect and update the data for dynamic analysis to ensure the predictive performance of the model.

## Conclusion

In the past 30 years, the U5MR and MMR of China have decreased significantly, and the gap between urban and rural areas has gradually narrowed. While it is still challenging to further reduce the U5MR and MMR with the adjustment of China’s birth policy and relatively low level of primary health services in some regions. The government should continue to promote the equalization of basic public health services, improve the accessibility and fairness of health care services, focus on the health status of children and pregnant women in rural areas, and further optimize the allocation of government health resources, so as to achieve the goals set by Healthy China by 2030. The relevant experience of China in reducing U5MR and MMR is of reference significance for the improvement of maternal and child health care in less developed countries. In the context of the Belt and Road Initiative, it is suggested that China’s advanced experience and applicable technologies in the field of maternal and child health be extended to developing countries to help achieve the global SDGs.

## Data availability statement

The datasets presented in this study can be found in online repositories. The names of the repository/repositories and accession number(s) can be found in the article/Supplementary material.

## Author contributions

MZ designed the study, conducted the data analysis, and drafted the main manuscript. HQ, JX, and XH contributed to the data collection. CS and FX contributed to writing the manuscript. JH and YC contributed to prepared figures and tables. HQ and MH provided technical support and guidance. All authors have read and agreed to the published version of the manuscript.

## Supplementary material

SUPPLEMENTARY TABLE 1 The data of neonatal mortality rate, infant mortality rate, under-five mortality rate and maternal mortality rate from 1991 to 2020 in China.

SUPPLEMENTARY TABLE 2 The maternal system management rate, prenatal care rate, postnatal visit rate, hospital delivery rate, GDP, total health expenditure and the ratio of total health expenditure to GDP from 1991 to 2020 in China.

SUPPLEMENTARY TABLE 3 The results of JoinPoint pairwise comparison test of NMR, IMR, U5MR and MMR between urban and rural areas.

SUPPLEMENTARY TABLE 4 The ARIMA predicted results of national, urban and rural areas from 2021-2025.

## References

[1] Bauserman M Thorsten VR Nolen TL Patterson J Lokangaka A Tshefu A. Maternal mortality in six low and lower-middle income countries from 2010 to 2018: risk factors and trends. Reprod Health. (2020) 17:173. doi: 10.1186/s12978-020-00990-z, PMID: 33334343 PMC7745363

[2] Golding N Burstein R Longbottom J Browne AJ Fullman N Osgood-Zimmerman A. Mapping under-5 and neonatal mortality in Africa, 2000-15: a baseline analysis for the sustainable development goals. Lancet. (2017) 390:2171–82. doi: 10.1016/S0140-6736(17)31758-0, PMID: 28958464 PMC5687451

[3] Hardelid P Davey J Dattani N Gilbert R. Child deaths due to injury in the four UK countries: a time trends study from 1980 to 2010. PLoS One. (2013) 8:e68323. doi: 10.1371/journal.pone.0068323, PMID: 23874585 PMC3707924

[4] Sachs JD McArthur JW. The millennium project: a plan for meeting the millennium development goals. Lancet. (2005) 365:347–53. doi: 10.1016/S0140-6736(05)17791-5 15664232

[5] Nielsen HS Eggebø TM. Millennium development goal 5--an obstetric challenge. Acta Obstet Gynecol Scand. (2012) 91:1007–8. doi: 10.1111/j.1600-0412.2012.01505.x 22897734

[6] Lozano R Wang H Foreman KJ Rajaratnam JK Naghavi M Marcus JR. Progress towards millennium development goals 4 and 5 on maternal and child mortality: an updated systematic analysis. Lancet. (2011) 378:1139–65. doi: 10.1016/S0140-6736(11)61337-8, PMID: 21937100

[7] Tan X Kong S Shao H. New strategies to improve the health of Chinese people by 2030. Aust J Prim Health. (2017) 23:307–8. doi: 10.1071/PY16146, PMID: 28803612

[8] GBD 2017. Causes of death collaborators. Global, regional, and national age-sex-specific mortality for 282 causes of death in 195 countries and territories, 1980-2017: a systematic analysis for the global burden of disease study. Lancet. (2017) 392:1736–88. doi: 10.1016/S0140-6736(18)32203-7, PMID: 30496103 PMC6227606

[9] Tan X Zhang Y Shao H. Healthy China 2030, a breakthrough for improving health. Glob Health Promot. (2019) 26:96–9. doi: 10.1177/1757975917743533, PMID: 29297762

[10] Zeng Y Hesketh T. The effects of China's universal two-child policy. Lancet. (2016) 388:1930–8. doi: 10.1016/S0140-6736(16)31405-2, PMID: 27751400 PMC5944611

[11] Li H Nawsherwan FC Yin S Haq IU Mubarik S Nabi G. Changes in adverse pregnancy outcomes in women with advanced maternal age (AMA) after the enactment of China's universal two-child policy. Sci Rep. (2022) 12:5048. doi: 10.1038/s41598-022-08396-6, PMID: 35322808 PMC8943149

[12] Chen Y Wu Y Dill S-E Guo Y Westgard CM Medina A. Effect of the mHealth-supported healthy future programme delivered by community health workers on maternal and child health in rural China: study protocol for a cluster randomised controlled trial. BMJ Open. (2023) 13:e065403. doi: 10.1136/bmjopen-2022-065403, PMID: 36669837 PMC9872510

[13] Chen Y Sylvia S Dill S-E Rozelle S. Structural determinants of child health in rural China: the challenge of creating health equity. Int J Environ Res Public Health. (2022) 19:13845. doi: 10.3390/ijerph192113845, PMID: 36360724 PMC9654689

[14] Zhao Q Chen J Li F Li A Li Q. An integrated model for evaluation of maternal health care in China. PLoS One. (2021) 16:e0245300. doi: 10.1371/journal.pone.0245300, PMID: 33507961 PMC7842919

[15] Zhang T Lu W Tao H. Efficiency of health resource utilisation in primary-level maternal and child health hospitals in Shanxi Province, China: a bootstrapping data envelopment analysis and truncated regression approach. BMC Health Serv Res. (2020) 20:179. doi: 10.1186/s12913-020-5032-y, PMID: 32143651 PMC7059375

[16] National Bureau of Statistics. The under-five and maternal mortality of surveillance region. (2022). Available at: https://data.stats.gov.cn/easyquery.htm?cn=B01.

[17] Zhao P Diao Y You L Wu S Yang L Liu Y. The influence of basic public health service project on maternal health services: an interrupted time series study. BMC Public Health. (2019) 19:824. doi: 10.1186/s12889-019-7207-1, PMID: 31242879 PMC6595598

[18] Zhao P Han X You L Zhao Y Yang L Liu Y. Effect of basic public health service project on neonatal health services and neonatal mortality in China: a longitudinal time-series study. BMJ Open. (2020) 10:e034427. doi: 10.1136/bmjopen-2019-034427, PMID: 32690734 PMC7375510

[19] Chen P Li M Zhu J Wang Y Mu Y Li Q. Provincial-level outcomes of China's 'Reducing maternal mortality and eliminating neonatal tetanus' program. Sci Rep. (2020) 10:13328. doi: 10.1038/s41598-020-70257-x, PMID: 32770045 PMC7414118

[20] Feng XL Shi G Wang Y Xu L Luo H Shen J. An impact evaluation of the safe motherhood program in China. Health Econ. (2010) 19:69–94. doi: 10.1002/hec.1593 20803630

[21] Jabbari Beyrami H Doshmangir L Ahmadi A Asghari Jafarabadi M Khedmati Morasae E Gordeev VS. Impact of rural family physician programme on maternal and child health indicators in Iran: an interrupted time series analysis. BMJ Open. (2019) 9:e021761. doi: 10.1136/bmjopen-2018-021761, PMID: 30647030 PMC6340439

[22] Li X Krumholz HM Yip W Cheng KK De Maeseneer J Meng Q. Quality of primary health care in China: challenges and recommendations. Lancet. (2020) 395:1802–12. doi: 10.1016/S0140-6736(20)30122-7, PMID: 32505251 PMC7272159

[23] Yip W Fu H Chen AT Zhai T Jian W Xu R. 10 years of health-care reform in China: progress and gaps in universal health coverage. Lancet. (2019) 394:1192–204. doi: 10.1016/S0140-6736(19)32136-1 31571602

[24] Mohammadi Y Parsaeian M Mehdipour P Khosravi A Larijani B Sheidaei A. Measuring Iran's success in achieving millennium development goal 4: a systematic analysis of under-5 mortality at national and subnational levels from 1990 to 2015. Lancet Glob Health. (2017) 5:e537–44. doi: 10.1016/S2214-109X(17)30105-5, PMID: 28363513

[25] United National Children’s fund. The state of the World's children. New York: UNICEF (2021).

[26] Cao H Wang J Li Y Li D Guo J Hu Y. Trend analysis of mortality rates and causes of death in children under 5 years old in Beijing, China from 1992 to 2015 and forecast of mortality into the future: an entire population-based epidemiological study. BMJ Open. (2017) 7:e015941. doi: 10.1136/bmjopen-2017-015941, PMID: 28928178 PMC5623503

[27] Liu Z Liu XR He CH Miao L Kang LN Li XH. Analysis of mortality and leading causes of death in Chinese children under 5-year-old between 2010 and 2016. Zhonghua Yu Fang Yi Xue Za Zhi. (2019) 53:411–4. doi: 10.3760/cma.j.issn.0253-9624, PMID: 30982278

[28] Cui H He C Kang L Li Q Miao L Shen L. Under-5-years child mortality due to congenital anomalies: a retrospective study in urban and rural China in 1996-2013. Am J Prev Med. (2016) 50:663–71. doi: 10.1016/j.amepre.2015.12.013, PMID: 26895742

[29] He C Liu L Chu Y Perin J Dai L Li X. National and subnational all-cause and cause-specific child mortality in China, 1996-2015: a systematic analysis with implications for the sustainable development goals. Lancet Glob Health. (2017) 5:e186–97. doi: 10.1016/S2214-109X(16)30334-5, PMID: 28007477 PMC5250590

[30] Xu XH Dong H Li L Liu WH Lin GZ Ou CQ. Trends and seasonality in cause-specific mortality among children under 15 years in Guangzhou, China, 2008-2018. BMC Public Health. (2020) 20:1117. doi: 10.1186/s12889-020-09189-0, PMID: 32678015 PMC7364532

[31] Shan D Qiu PY Wu YX Chen Q Li AL Ramadoss S. Pregnancy outcomes in women of advanced maternal age: a retrospective cohort study from China. Sci Rep. (2018) 8:12239. doi: 10.1038/s41598-018-29889-3, PMID: 30115949 PMC6095911

[32] Wu Y Chen Y Shen M Guo Y Wen SW Lanes A. Adverse maternal and neonatal outcomes among singleton pregnancies in women of very advanced maternal age: a retrospective cohort study. BMC Pregnancy Childbirth. (2019) 19:3. doi: 10.1186/s12884-018-2147-9, PMID: 30606150 PMC6318893

[33] Chen L Feng P Shaver L Wang Z. Maternal mortality ratio in China from 1990 to 2019: trends, causes and correlations. BMC Public Health. (2021) 21:1536. doi: 10.1186/s12889-021-11557-3, PMID: 34380436 PMC8359022

[34] Liu J Song L Qiu J Jing W Wang L Dai Y. Reducing maternal mortality in China in the era of the two-child policy. BMJ Glob Health. (2020) 5:e002157. doi: 10.1136/bmjgh-2019-002157, PMID: 32133196 PMC7042574

[35] Harpur A Minton J Ramsay J McCartney G Fenton L Campbell H. Trends in infant mortality and stillbirth rates in Scotland by socio-economic position, 2000-2018: a longitudinal ecological study. BMC Public Health. (2021) 21:995. doi: 10.1186/s12889-021-10928-0, PMID: 34044796 PMC8155799

[36] Fell DB Park AL Sprague AE Islam N Ray JG. A new record linkage for assessing infant mortality rates in Ontario, Canada. Can J Public Health. (2020) 111:278–85. doi: 10.17269/s41997-019-00265-6, PMID: 31858437 PMC7109219

[37] Galan J Mydam J Collins JW. Infant mortality rates among US-born and foreign-born Latinx women. The effect of black race. Matern Child Health J. (2022) 26:511–6. doi: 10.1007/s10995-021-03366-2, PMID: 35199230

[38] Nath S Hardelid P Zylbersztejn A. Are infant mortality rates increasing in England? The effect of extreme prematurity and early neonatal deaths. J Public Health. (2021) 43:541–50. doi: 10.1093/pubmed/fdaa025 PMC8458015 32119086

