# Supplementary material for: Trends, influencing factors and prediction analysis of under-five and maternal mortality rates in China from 1991 to 2020
Source: Front Public Health. 2023 Oct 19;11:1198356. doi: 10.3389/fpubh.2023.1198356 (PMC10620530; doi:10.3389/fpubh.2023.1198356)
Supplement: SUPPLEMENTARY TABLE 3 — The results of JoinPoint pairwise comparison test of NMR, IMR, U5MR and MMR between urban and rural areas. [file Table_3.DOCX]

|  | **Range** | **Lower Endpoint** | **Upper Endpoint** | **AAPC Difference**  **(%)** | **Lower CI**  **(%)** | **Upper CI**  **(%)** | **Test Statistic** | **P-Value** |
| --- | --- | --- | --- | --- | --- | --- | --- | --- |
| NMR | Full Range | 1991 | 2020 | 1.4 | -0.7 | 3.6 | 1.7 | 0.193 |
| IMR | Full Range | 1991 | 2020 | 1.8 | 0.8 | 2.8 | 3.5 | < 0.001 |
| U5MR | Full Range | 1991 | 2020 | 1.6 | 0.5 | 2.7 | 2.9 | 0.003 |
| MMR | Full Range | 1991 | 2020 | 2.1 | 0.6 | 3.6 | 2.8 | 0.005 |

The results of JoinPoint pairwise comparison test of NMR, IMR, U5MR and MMR between urban and rural areas.
